# MicroRNA-148b regulates tumor growth of non-small cell lung cancer through targeting MAPK/JNK pathway

**DOI:** 10.1186/s12885-019-5400-3

**Published:** 2019-03-08

**Authors:** Lin Lu, Qiyao Liu, Peipei Wang, Yong Wu, Xia Liu, Chengyin Weng, Xisheng Fang, Baoxiu Li, Xiaofei Cao, Haibo Mao, Lina Wang, Mingmei Guan, Wei Wang, Guolong Liu

**Affiliations:** 1 Department of Medical Oncology, Guangzhou First People’s Hospital, Guangzhou Medical University, Guangzhou, 510180 Guangdong China; 2 Department of Medical Oncology, Guangzhou First People’s Hospital, School of Medicine, South China University of Technology, Guangzhou, 510180 Guangdong China; 3 Department of Experimental Research and State Key Laboratory of Oncology in Southern China, Sun Yat-Sen University Cancer Center, Guangzhou, 510080 Guangdong China

**Keywords:** microRNA-148b, Non small cell lung cancer (NSCLC);tumor suppressor, Proliferation, Apoptosis, MAPK/JNK pathway

## Abstract

**Background:**

MicroRNA-148b (miR-148b) has been detected in various types of tumors, and is generally viewed as a tumor suppressor. Our previous study found the decreased expression of miR-148b in human non small cell lung cancer (NSCLC) specimens and cell lines. However, the underlying mechanisms of miR-148b in regulating tumor progression remain unclear.

**Methods:**

Firstly animal experiments were performed to verify whether miR-148b could inhibit the tumor growth. Then, the underlying mechanisms were studied by transfecting recombinant plasmids containing a miR-148b mimic or a negative control (NC) mimic (shRNA control) into NSCLC cell lines PC14/B and A549 cells. Tumor cells transfected with unpackaged lentiviral vectors was used as blank control. Cell proliferation capabilities were measured by using CCK-8 kit and colony formation assay. Cell cycle arrest was compared to clarify the mechanism underlying the tumor cell proliferation. Annexin V-FITC Apoptosis Detection kit was applied to investigate the effect of miR-148b on cell apoptosis. Furthermore, western blot analysis were performed to study the targeting pathway.

**Results:**

We found that over-expression of miR148b could significantly inhibit tumor growth, while knocking down miR148b could obviously promote tumor growth. Further experiment showed that miR-148b inhibited tumor cell proliferation. Besides, over-expression of miR148b decreased the G2/M phase population of the cell cycle by preventing NSCLC cells from entering the mitotic phase and enhanced tumor cell apoptosis. Further western blot analysis indicated that miR148b could inhibit mitogen-activated protein kinase/Jun N-terminal kinase (MAPK/JNK) signaling by decreasing the expression of phosphorylated (p) JNK.

**Conclusions:**

These results demonstrate that miR-148b could inhibit the tumor growth and act as tumor suppressor by inhibiting the proliferation and inducing apoptosis of NSCLC cells by blocking the MAPK/JNK pathway.

## Background

Lung cancer is the most frequent cause of cancer-related deaths worldwide. Each year, 1.8 million people are diagnosed with lung cancer, and 1.6 million people die from this disease. Five-year survival rates of lung cancer patients vary from 4 to 17%, depending on their tumor stages and regional differences [1]. Lung cancer are usually classified into non-small cell lung cancer (NSCLC) and small-cell lung cancer (SCLC) depending on their pathological and histological characteristics [2]. Most NSCLC patients were present with metastatic disease at the time of diagnosis [3]. The brain or central nervous system is a common metastatic site for NSCLC, with 40–50% of patients developing brain metastasis [4–6]. Patients with NSCLC and brain metastases have a poor prognosis, with a median overall survival between 4 and 9 months with chemotherapy, and only 7 months for patients receiving whole-brain radiation therapy [7]. Therefore, innovative therapeutic approaches held great promise in the management of NSCLC.

MicroRNAs (miRNAs) are single stranded small non-coding RNAs [8]. By completely or incompletely binding with the 3′-untranslated region of the miRNA (3′-UTR), miRNAs regulate protein expression [9–11]. Abnormal expression of miRNAs may lead to alterations in processes that are important in the tumor development, including cellular differentiation, proliferation, apoptosis and metastasis [12–14]. Numerous studies have proofed that the structures of artificial miRNAs and endogenous miRNAs are similar and will not cause an endogenous reaction [15]. Thus, miRNA therapy is considered a potentially safe and promising treatment method for cancer patients [16].

MiR-148b has been detected downregulated in several types of human tumors, including hepatocellular carcinoma [17, 18], chronic myeloid leukemia [19], breast cancer [20, 21], lung cancer [22], pancreatic cancer [23], gastric cancer [24] and colorectal cancer [25]. Our previous study also detected decreased expression of miR-148b in human non small cell lung cancer (NSCLC) tissues and cell lines [22]. However, the contribution of miR-148b to tumor progression and its potential mechanisms have not been fully explored. To better understand the biological significance of miR-148b in NSCLC, we firstly performed animal experiments to verify the tumor suppressor function of miR-148. Then, we studied the effect of miR-148b in tumor cell proliferation and apoptosis, and target signal pathway was investigated.

## Methods

### Cell culture and transfection

Human non small cell lung cancer cell lines PC14/B and A549 were purchased from the Committee of the Type Culture Collection of the Chinese Academy of Sciences, Shanghai, China in the year of 2016. Cell lines cultured in RPMI-1640 medium (Gibco, Thermo Fisher Scientific, USA) supplemented with 10% fetal bovine serum (Gibco, Thermo Fisher Scientific, USA). The identity of the cell lines have been validated by short-tandem repeat analyses. They were free of mycoplasma contamination. The 293 T normal renal cell line was maintained in Dulbecco’s modified Eagle’s medium (Gibco, Thermo Fisher Scientific) supplemented with 10% FBS (18). MiR-148b mimic (UCAGUGCAUCACAGAACUUUGU) and negative control (NC) mimic (shRNA) were synthesized by RiboBio Co. (China). miR-148b mimic was used to construct the lentiviral rLv-miR-148b vector as a miR-148b overexpression group, and NC mimic was used to construct lentiviral rLv-shRNAvector to interfere with the expression of miR-148b as a negative control group. The lentiviral vector was packaged by co-transfection of 293 T cells with a recombinant plasmid containing a miR-148b mimetic or a negative control mock. After concentration and purification of the lentiviral vector, the titer of the miR-148b lentiviral vector is determined to be 1.25X10^8^ transducing units(TU)/ml, and the titer of the NC lentiviral is 1.5X10^8^ transducing units(TU) /ml. The multiplicity of infection(MOI) was determined to be 50 based on the effect of cell infection. The viral vector rLv-miR-148b or the viral vector rLv-NC was then stably transfected into PC14/B and A549 cells by Lipofectamine TM2000 according to the manufacture’s instructions. The blank group was transfected with the unrecombined lentiviral vector rLv-Blank at the same MOI. The puromycin resistance gene carried together in the lentiviral vector is used for cell screening by puromycin drugs after stable cell transfection. After the stable transfectant was successfully constructed, it was continuously cultured using a low concentration of puromycin-containing medium to ensure stable cell transfection.

### Mice

Female BALB/c nude mice were supplied by the Laboratory Animals Center of Sun Yat-Sen University. The mice were housed in specific pathogen-free condition at the animal facilities of South China University of Technology. Guangzhou First People’s Hospital approved all animal protocols.

### Tumorigenicity

The mice at the age of 6 to 8 weeks were randomly divided into three groups and each group contained at least 8 mice. PC14/B (1 × 10^6^/mouse in 0.2 ml of PBS) and A549 cells (2 × 10^6^/mouse in 0.2 ml of PBS) were subcutaneously inoculated into the right flank of the nude mice. The tumor size was measured every 3 days by measuring the length (L) and width (W). And the tumor volume was calculated using formula: (LxW2)/2. After 6 week implantation and the last measurement of tumor volume, animals were euthanized by carbon dioxide inhalation followed by cervical dislocation and the subcutaneous tumors were collected and weighted.

### Quantitative real-time PCR (qRT-PCR)

Total RNA was extracted using TRIzol (Invitrogen, USA) according to the manufacturer’s protocol. cDNA was synthesized with the MLV transcriptase Kit (Invitrogen, USA). The quantitative analysis of miR-148b expression was assayed using a Bulge-Loop TM miRNA qRT-PCR primer (forward, 5′-ACACTCCAGCTGGGTCAGTGCATC-3′ and reverse, 5′-CTCAACTGGTGTCGTGGA-3′; RiboBio, China) and Platinum® SYBR® Green qPCR SuperMix-UDG with ROX (Invitrogen, USA) on an ABI PRISM® 7500 Sequence Detection System (Applied Biosystems, Foster, CA). U6 small nuclear RNA (forward, 5′- CTCGCTTCGGCAGCACA-3′ and reverse, 5′-AACGCTTCACGAATTTGCGT-3′) was used as an internal control (RiboBio, China). The fold changes were calculated through relative quantification using the 2-ΔΔCt method (18).

### Western blot analysis

Proteins were extracted from PC14/B and A549 cells using RIPA buffer (Beyotime Biotechnology Company, China). Equal amounts (20 μg) of protein lysate were separated on parallel lanes of a 12% SDS PAGE gel and then electrotransferred onto a PVDF membrane (Millipore, USA. Following blocking with 5% non-fat milk in PBST (phosphate-buffered saline containing Tween 20) for 1 h, the membranes were incubated overnight at 4 °C with the following antibodies: phosphor (p)-JNK (Thr183/Tyr185) rabbit polyclonal antibody (1:500 dilution; Immuno Way, USA), (t) JNK mouse monoclonal antibody (1:500 dilution; Proteintech, USA), mitogen activated protein kinase kinase 4 (MKK4) rabbit polyclonal antibody (1:200 dilution; Proteintech, USA), or mitogen activated protein kinase kinase 7 (MKK7) rabbit polyclonal antibody (1:200 dilution; Proteintech, USA). Glyceraldehyde 3-phosphate dehydrogenase (GAPDH) rabbit monoclonal antibody (1:10,000 dilution; Abcam, USA) was used as a protein loading control. After being washed thrice with PBST, the membranes were incubated with the corresponding secondary antibody HRP-conjugated goat anti-mouse immunoglobulin (Ig)G (1:2000 dilution; Abcam, USA). After being incubated for 1 h at room temperature, the protein bands were developed using an Immobilon Western Chemiluminescent HRP Substrate kit (Millipore, USA) and visualized using a ChemiDoc XRS+ Imaging system (Bio-Rad Laboratories, Inc., USA). Gray value analysis of protein bands was conducted for quantification of the protein levels by Image Lab software (Bio-Rad Laboratories, Inc., USA).

### Cell counting kit-8 (CCK-8) assay

Tumor cells (1 × 10^3^ cells per well) were seeded into 96-well plates in a final volume of 100 μl and incubated overnight at 37 °C. The proliferation of the treated cells was determined by adding 10 μl of CCK-8 per well (KeyGen Biotech, China). Following incubation at 37 °C for 4 h, the absorbance values of each well were measured at 450 nm using a microplate reader (Olympus, Japan).

### Colony formation assay

Tumor cells were seeded in 6-well plates at a concentration of 500 cells per well, and medium was replaced with fresh medium every 3 days as the general colony formation assay method [26, 27]. After cultivation for 14 days, the cells were washed and fixed using 75% ethanol for 15 min. Following staining with 0.5% crystal violet for 15 min, the cells were washed and air dried at room temperature. The cells were then visualized under a microscope, and colonies comprising 50 or more cells were counted.

### Cell cycle assay

PC14/B and A549 cells were rinsed thrice with cold PBS. Then the cells were collected and cell concentration were adjusted to 1 × 10^6^ /ml. 1 ml of single cell suspension were taken to perform the cell cycle assay. After removing the supernatant, the cells were fixed in cold 75% ethanol overnight at − 20 °C. After three rinses with cold PBS, the cells were treated with RNase A (KeyGen Biotech, China) in a 37 °C thermostat water bath for 30 min and stained with PI (KeyGen Biotech, China) in the dark at 4 °C for 30 min. Subsequently, the cells were analyzed by flow cytometry (BD Biosciences, USA).

### Cell apoptosis assay

The apoptotic cell rates were determined using the Annexin V-FITC Apoptosis Detection kit (KeyGen Biotech, China) according to the manufacturer’s instructions. Cells were rinsed twice with cold PBS and re-suspended in binding buffer. Following incubation with Annexin V-FITC reagent and PI in the dark at room temperature for 15 min, the cell suspension was analyzed by flow cytometry (BD Biosciences, USA).

### Statistical analysis

All of the data were analyzed using GraphPad Prism 6 software (GraphPad Inc., USA). Numerical data were expressed as the mean ± standard deviation (SD) of at least three independent experiments. Differences between two groups were determined by the Student’s t-test, and correlation analysis was evaluated by Pearson’s correlation. Statistical significance was considered when *P* < 0.05.

## Results

### Over-expression of miR-148b inhibited tumor growth

**Fig. 1:**
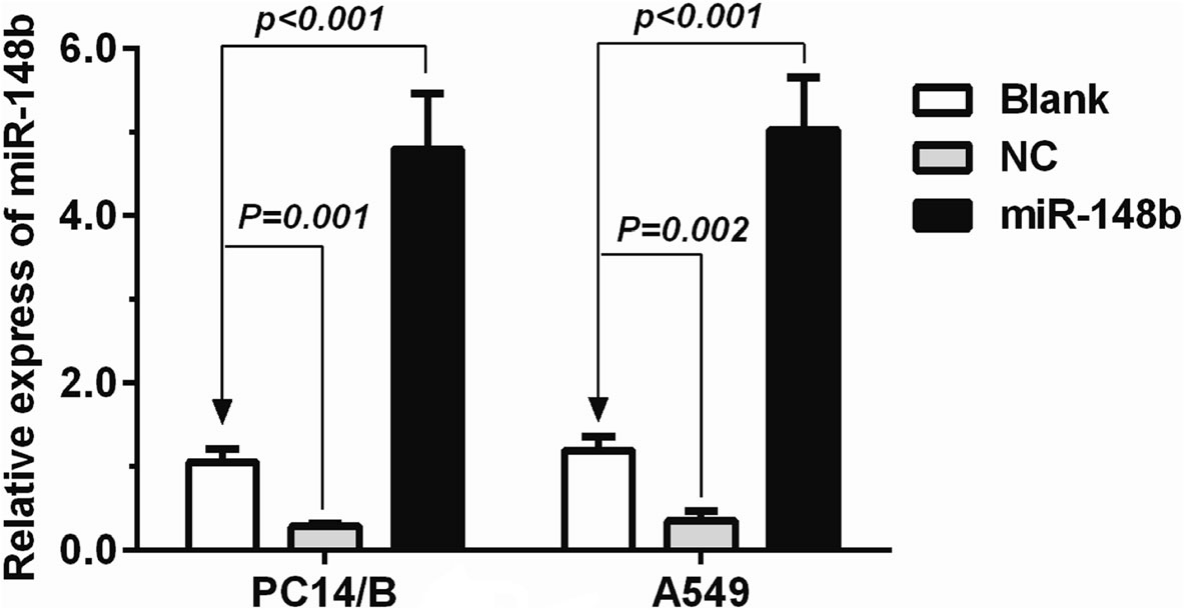
The expression of miR-148b analyzed using real time quantitative PCR (qRT-PCR). miR-148b mimics (miR-148b) or negative control mimics (NC, shRNA control) were stably transfected into non small cell lung cancer cell lines PC14/B and A549 cells. The blank groups (blank) were transfected with unpackaged lentiviral vectors at the same MOI. The expression of miR-148b in PC14/B and A549 cells were assessed by qRT-PCR. U6 RNA was used as an internal control

Our previous study detected decreased expression of miR-148b in human NSCLC tissues and cell lines [22], which indicating that miR-148b might work as a tumor suppressor in NSCLC. Thus, in this study, we further investigated the effect and underlying mechanisms of miR-148b in tumor progression. Firstly, miR-148b mimics or negative control mimics (shRNA control) were stably transfected into PC14/B and A549 cells. The blank groups were transfected with unpackaged lentiviral vectors at the same MOI. Changes in the expression of miR-148b in PC14/B and A549 cells were assessed by qRT-PCR (Fig. 1).

**Fig. 2:**
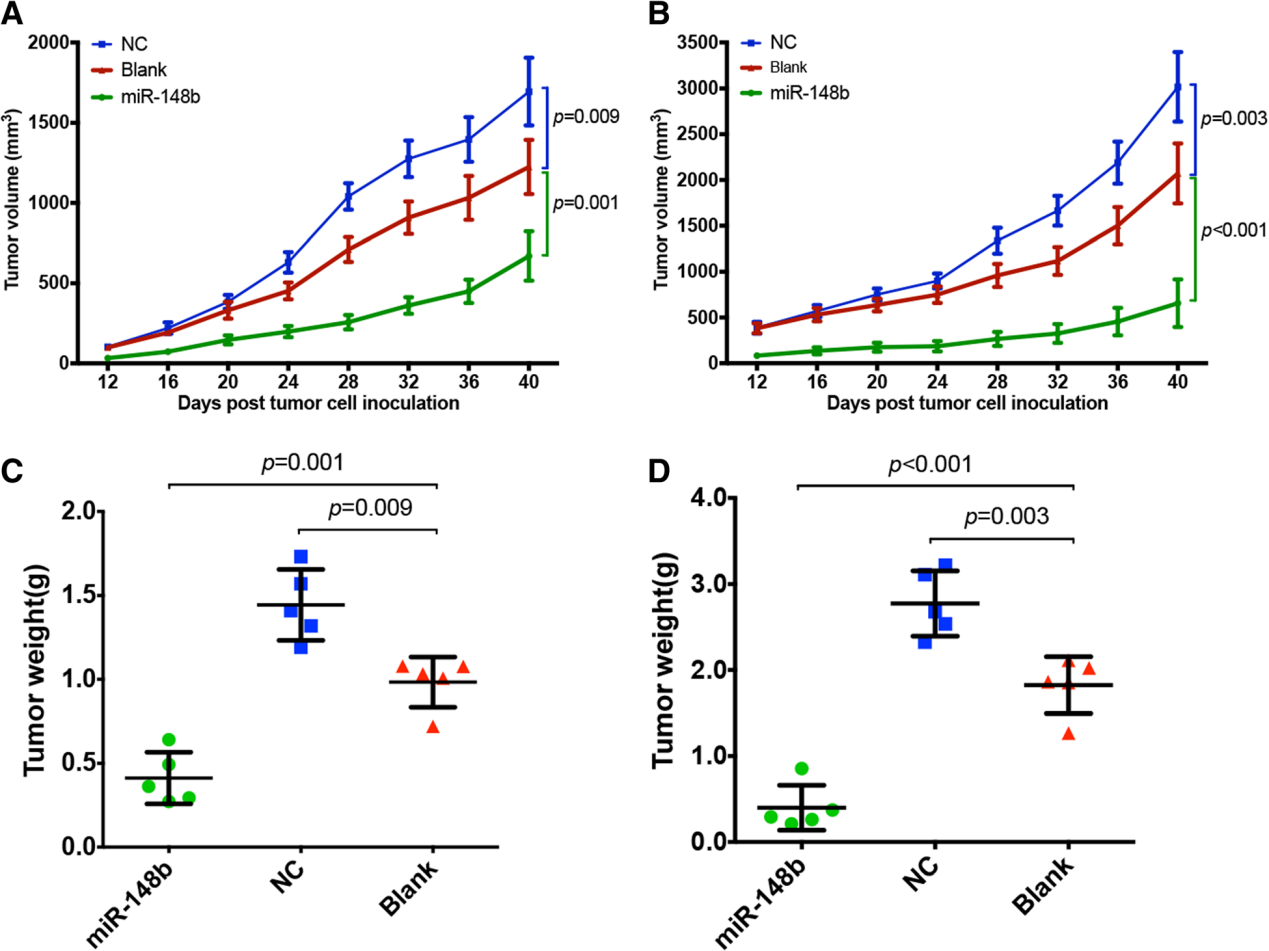
miR-148b inhibits the tumor growth. Female BALB/c nude mice were subcutaneously inoculated with PC14/B and A549 cells transfected with miR-148b mimics (miR-148b), negative control mimics (NC, shRNA control) or unpackaged lentiviral vectors (blank). Over-expression of miR-148b significantly inhibited local tumor growth both in PC14/B model (**a** and **c**) and A549 model (**b** and **d**)

We performed mouse models to verify our previous results that miR-148b might work as a tumor suppressor. As shown in Fig. 2, PC14/B and A549 cells transfected with miR-148b mimics developed much smaller local tumors than blank groups (*P* = 0.001 and *P* < 0.001, respectively, Fig. 2). Besides, knocking down miR-148b by transfecting tumor cells with a negative control (NC) mimic could significantly promote the tumor growth compared to blank groups (*P* = 0.009 and *P* = 0.003, respectively, Fig. 2). Same results were generated both in PC14/B (Fig. 2a) and A549 (Fig. 2b) models. These results provided evidence that miR-148b could be viewed as a tumor suppressor in NSCLC.

### The effect of miR-148b on the proliferation of NSCLC cells

**Fig. 3:**
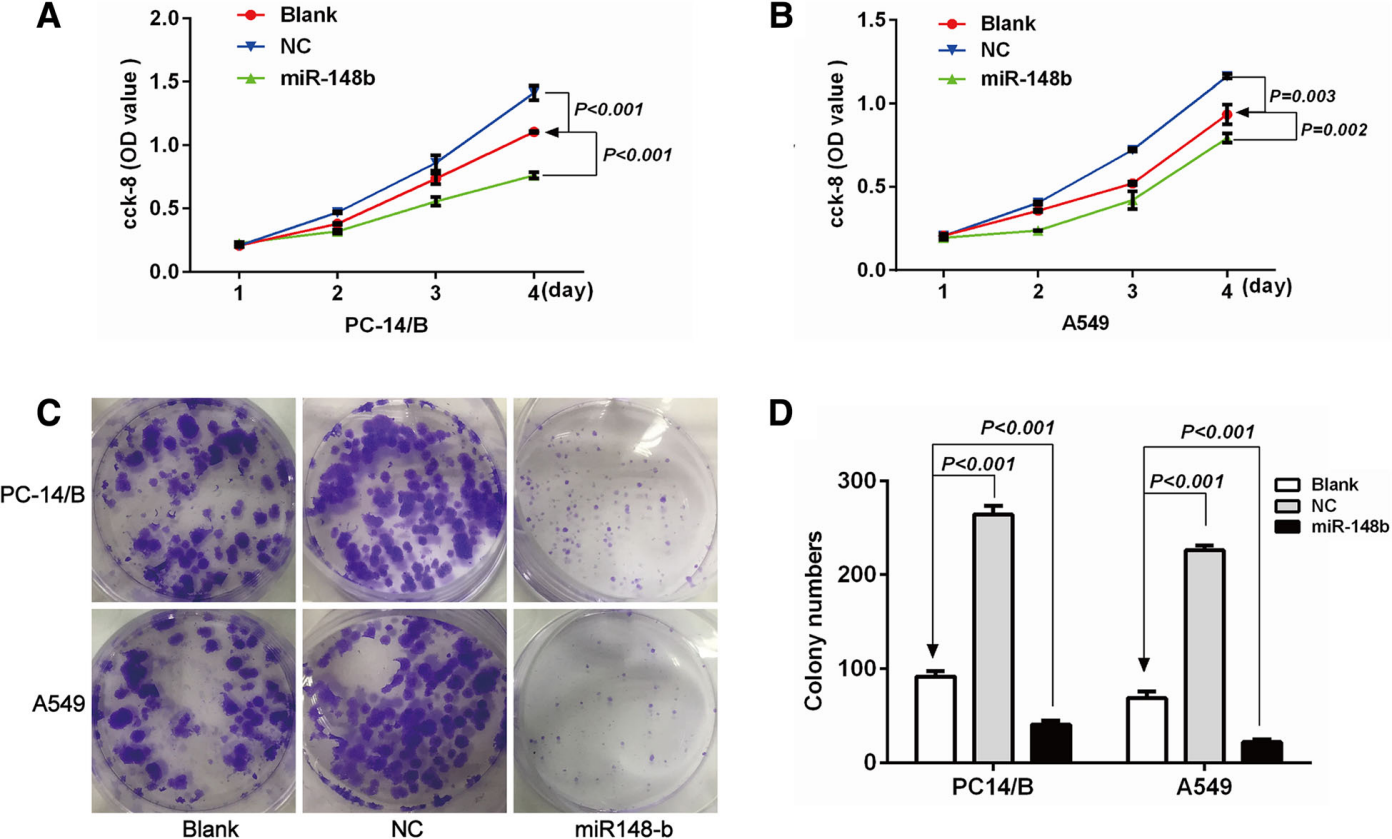
miR-148b inhibited the proliferation of NSCLC cells. The total number of viable PC14/B **a** and A549 cells **b** after being transfected with different mimics was assessed using a Cell Counting Kit-8 assay. The data are expressed as optical density (OD). **c** and **d** The colony formation assay for PC14/B and A549 cells. Transfected with different mimics. Over-expression of miR-148b significantly inhibited PC14/B and A549 cell proliferation and colony formation compared to the blank group. While knocking down miR-148b expression (NC) could reverse miR-148b-mediated proliferation and colony formation inhibiting effect in PC14/B and A549 cells. The results are expressed as the mean ± standard deviation. NC, negative control; miR, microRNA; Blank, the cells transfected with unpackaged lentiviral vectors; OD, optical density

It’s generally accepted that tumor cell proliferation and apoptosis play important roles in tumor development. Thus we further investigate the functional contribution of miR-148b in cell proliferation and apoptosis in NSCLC. As shown in Fig. 3a and b, over-expression of miR-148b significantly inhibited PC14/B (*P* < 0.001, Fig. 3a) and A549 cell (*P* = 0.002, Fig. 3b) proliferation compared to the blank group. And knocking down miR-148b expression through transfecting negative control mimics (NC) could reverse miR-148b-mediated proliferation (*P <* 0.001 for PC14/B and *P* = 0.001 for A549, respectively, Fig. 3a and b).

Representative graphs of colony formation were presented in Fig. 3c. Over-expression of miR-148b could significantly inhibit PC14/B and A549 cell colony formation compared to the blank group (*P* < 0.001, Fig. 3d). Besides, compared to the blank group, knocking down miR-148b expression by transfecting negative control mimics (NC) could significantly promoted the colony formation capabilities of PC14/B and A549 cells (*P* < 0.001, Fig. 3d). These results indicate that miR-148b could regulate the proliferation of NSCLC cells.

### The effect of miR-148b on the cell cycle of NSCLC

**Fig. 4:**
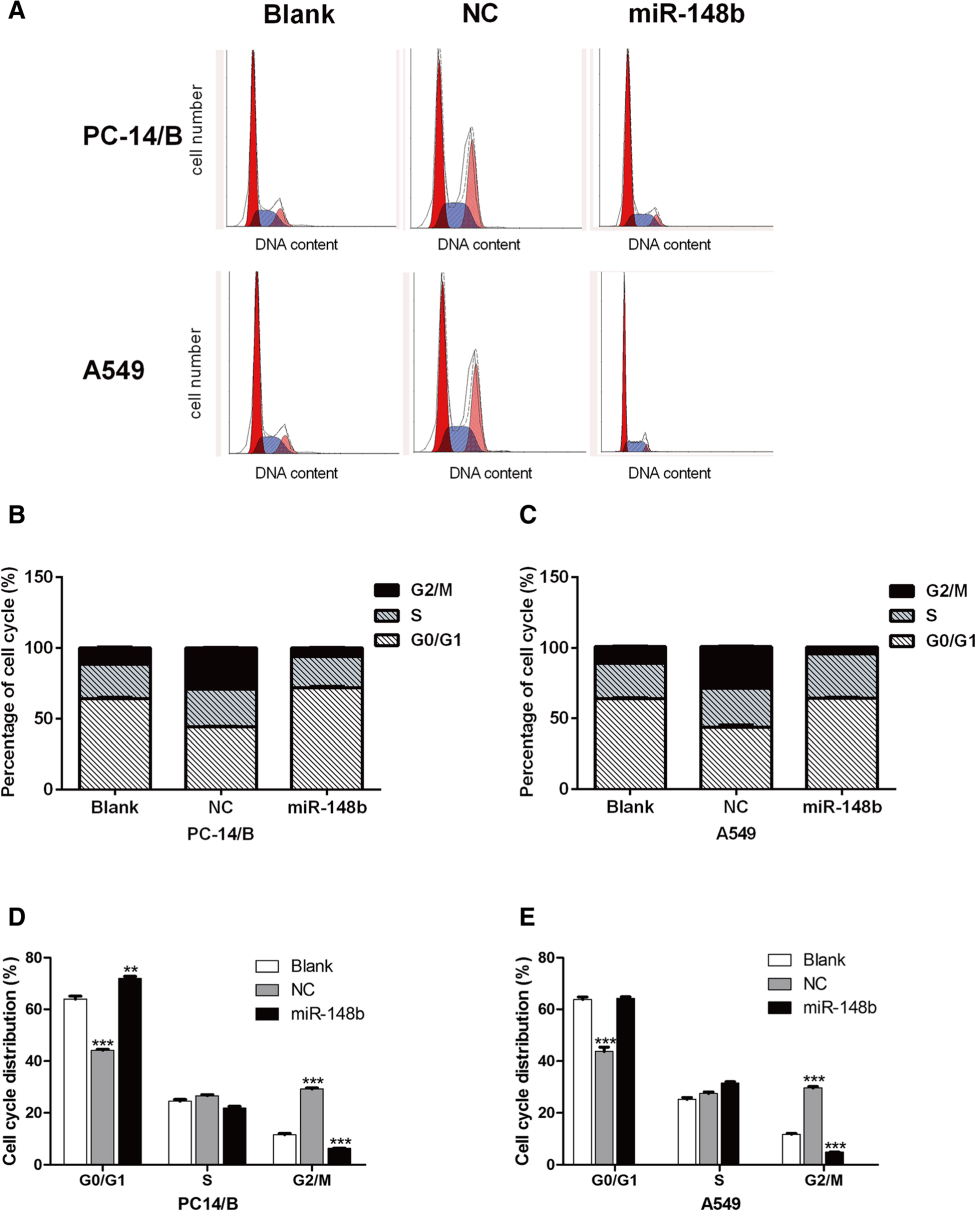
miR-148b blocks the cell cycle at the G2/M phase. **a** Cell cycle distribution of PC14/B and A549 cells after being transfected with different mimics. Red color represents G0/G1 phase, pink color represents G2/M phase, blue color represents S phase. **b** and **d** The percentage of cell cycle distribution in PC14/B cells. **c** and **e** The percentage of cell cycle distribution in A549 cells. The results are expressed as the mean ± standard deviation. Blank, cells transfected with unpackaged lentiviral vectors; NC, negative control; miR, microRNA

To explore the effect of miR-148b on the cell cycle of NSCLC cells, we examined the cell cycle of PC14/B and A549 cells after being transfected with different mimics. The cell population in G2/M phase was significantly decreased in PC14/B (Fig. 4a, b and d) and A549 (Fig. 4a, c and e) cells after being transfected with miR-148b mimics compared to the blank group, while the G2/M phase was significantly increased after being transfected with negative control mimics. These results indicated that miR-148b suppressed NSCLC cell entry to the mitotic phase, although it had no effect on DNA replication activity (S phase). These data suggest that miR-148b could work on the cell cycle.

### The effect of miR-148b on the apoptosis of NSCLC cells

**Fig. 5:**
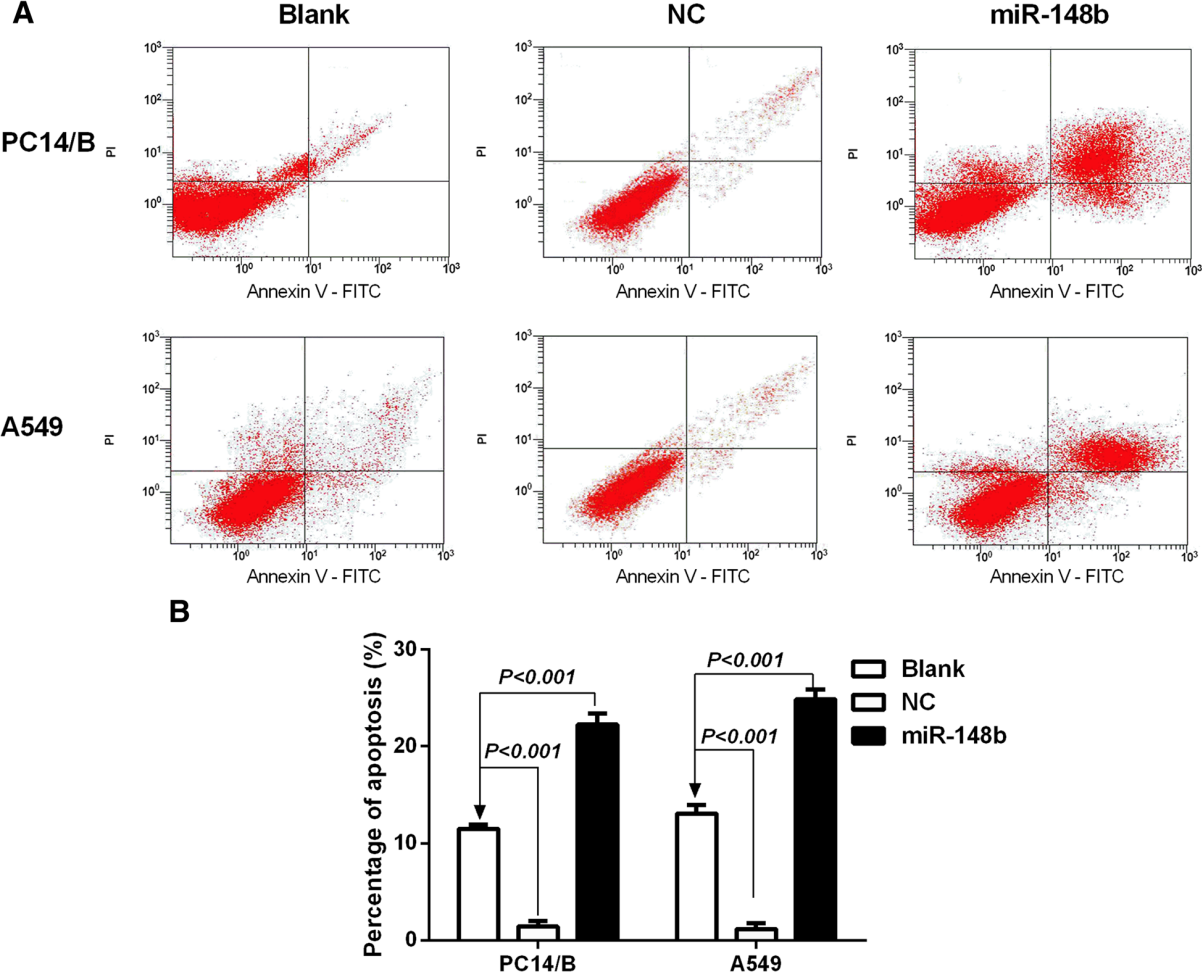
miR-148b induces apoptosis in NSCLC cells*.* **a** Annexin V-FITC/PI double staining and flow cytometric analysis was applied to detect the apoptotic rate of PC14/B and A549 cells after being transfected with different mimics. **b** Early apoptotic rates obtained by quantification of results generated from graph A. the apoptotic rate of the miR-148b-overexpression cells was higher than the blank group, while the apoptotic rate of the miR-148b-lowexpression cells was below the blank group. The results are expressed as the mean ± standard deviation. PI, propidium iodide; Blank, cells transfected with unpackaged lentiviral vectors; NC, negative control; miR, microRNA

To examine whether miR-148b influences the apoptotic rate of PC14/B and A549 cells, Annexin V-FITC/propidium iodide (PI) double staining and flow cytometric analysis were conducted. As demonstrated in Fig. 5a, over-expression of miR-148b could induce apoptosis of tumor cell. The apoptotic rate of the miR-148b-overexpression cells was higher than the blank groups (*P* < 0.001, Fig. 5b). While the apoptotic rate of the miR-148b low-expression cells (NC group) was l lower than the blank group (*P* < 0.001, Fig. 5b). These date indicate that miR-148b could work on cell apoptosis.

### miR-148b inhibited the MAPK/JNK signaling pathway by decreasing the expression of phosphorylated (p) JNK in NSCLC cells

**Fig. 6:**
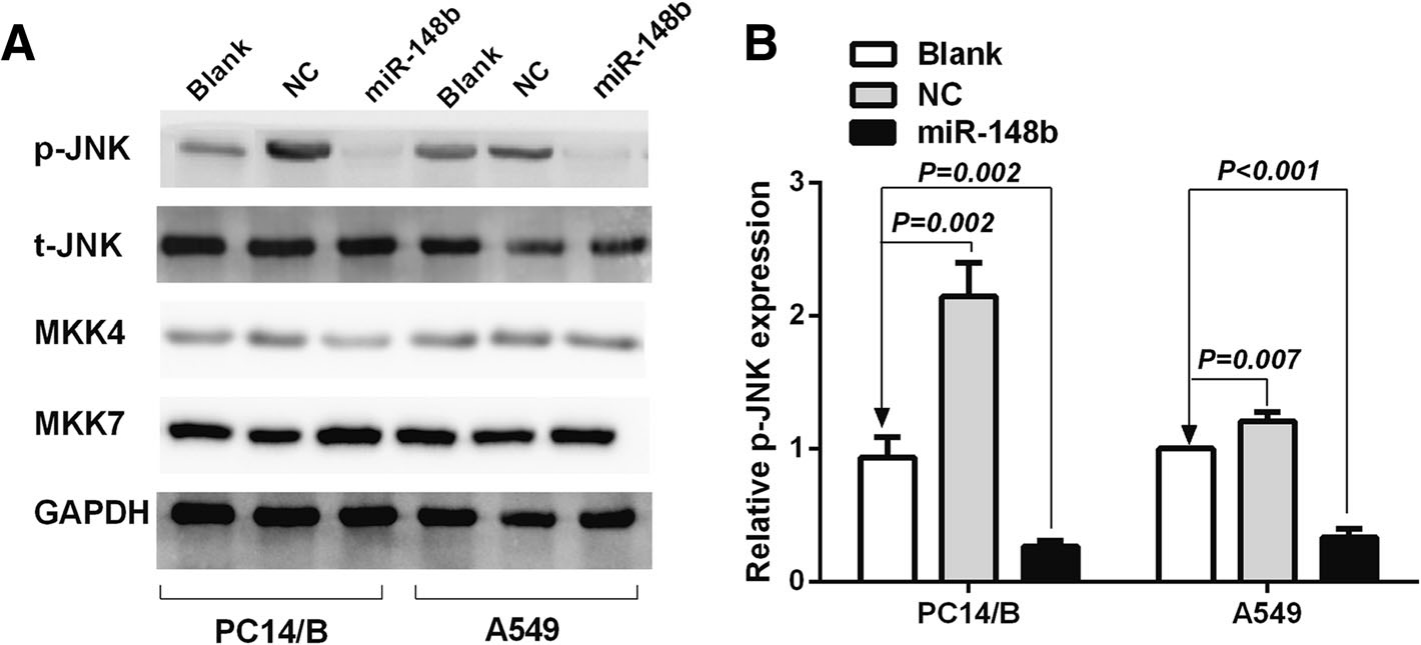
miR-148b inhibits the MAPK/JNK signaling pathway by decreasing the expression of phosphorylated (p) JNK in NSCLC cells. **a** Western blot analysis of phosphorylated(p) JNK, unphosphorylated (t) JNK, MKK4, MKK7 and GAPDH protein expression in PC14/B and A549 cells after being transfected with different mimics. **b** Protein bands were relative quantified by Image Lab software analysis. Over-expression of miR-148b obviously decreased the protein expression of phosphorylated JNK in PC14/B and A549 cells compared to the blank group, while the protein expression of MKK4, MKK7 and unphosphorylated (t) JNK was not affected. Phosphorylation of JNK was reactivated by knocking down miR-148b expression. The results are expressed as the mean ± standard deviation. JNK, c-Jun N-terminal kinase; p-JNK, phosphorylated JNK; t-JNK, unphosphorylated JNK; MKK4, mitogen-activated protein kinase kinase 4; MKK7, mitogen-activated protein kinase kinase 7; GAPDH, Glyceraldehyde 3-phosphate dehydrogenase, loading control protein; Blank, the cells were transfected with unpackaged lentiviral vectorsI; NC, negative control; miR, microRNA

It is well known that JNK belongs to the MAPK family. JNK activity regulates several important cellular functions, including cell proliferation, differentiation, survival and apoptosis. It is activated by dual phosphorylation of MKK4 and MKK7. Therefore, we further investigated the role of MAPK/JNK signaling pathway in regulating the proliferation and apoptosis of miR-148b in NSCLC. Representative western blot results were demonstrated in Fig. 6a. Over-expression of miR-148b obviously decreased the protein levels of phosphorylated JNK in PC14/B and A549 cells compared to the blank group (*P* = 0.002 and *P* < 0.001, respectively, Fig. 6b), while the protein levels of MKK4, MKK7 and unphosphorylated (t) JNK was not affected. Phosphorylation of JNK was re-activated by knocking down miR-148b expression (*P* = 0.002 and *P* = 0.007, respectively, Fig. 6b). Our results showed that miR-148b could inhibit the MAPK/JNK signaling pathway by decreasing the expression of phosphorylated (p) JNK in NSCLC cells.

## Discussion

It is generally accepted that during cancer progression, multiple oncogene proteins are up-regulated. In contrast, numerous tumor-suppressing proteins are down-regulated. The mechanisms of regulating changes in protein expression are very complex. Among these tumor promoting and suppressive proteins, miRNAs have been recognized to play an important role in epigenetic changes [28, 29]. Dysregulated miRNAs expression takes part in multiple biological processes, including regulation of oncogenes and suppressor genes expression, tumor progression and metastasis [30, 31]. Down-regulation of miR-148b has been identified in various types of human cancer, and miR-148b was regarded as a tumor-suppressive miRNA [21, 32, 33]. The lncRNAs (long non-coding RNAs) controlled by miR-148b are thought to play an important role in immune system and are associated with the development and progression of gastric cancer [34]. MiR-148b was reported to increase the radio-sensitivity of non-Hodgkin’s cells in non-Hodgkin’s Lymphoma [35]. Additionally, miR-148b exerted an effect on cell proliferation by regulating the expression of the cholecystokinin-2 receptor gene in colorectal cancer [18]. These findings indicated that dysregulation of miR-148b closely related to malignancies. Besides, our previous studies found that miR-148b was down-regulated in NSCLC tissues, suggesting that miR-148b might participate in the malignant degeneration of NSCLC.

In this study, we further investigate the tumor suppressive functions and underlying mechanisms of miR-148b in NSCLC by both over-expressing and knocking down the expression of miR-148b in NSCLC cell lines. Firstly, we carried out mouse model to verify the tumor suppressive function of miR-148b. Our research found that over-expression of miR-148b could significantly inhibited tumor growth, while knocking down miR-148b significantly promoted tumor growth. There were positive correlations between the xenograft model, the CCK8 assay, and the colony formation assay. When the CCK8 assay of the miR48b group demonstrated a small OD value, the corresponding colony formation was smaller, and the volume of the subcutaneous tumor of the mouse was smaller. In contrast, when the CCK8 assay demonstrated a large OD value in the NC group, the colony formation of the NC group was larger, and the volume of the subcutaneous tumor was larger. These results combined with our previous study revealed that miR-148b could be viewed as a tumor suppressor in NSCLC [22]. Consistently, Ge et al. detected decreased expression of miR-148b in NSCLC tumor tissues, and decreased expression of miR-148b was correlated with poor survival [36]. Li et al. detected the expression of miR-148b in serum of NSCLC patients, and they found that downregulation of miR-148b was correlated with more aggressive tumors [37].

We further studied the mechanisms underlying the miR-148b-mediated anti-cancer process, and found that over-expression of miR-148b could significantly inhibit NSCLC cell proliferation, colony formation, and induce cell apoptosis. Knocking down the miR-148b expression could reverse these effects. Over-expression of miR-148b significantly decreased the G2/M phase population in NSCLC cells by preventing NSCLC cells from entering the mitotic phase to inhibit the cell proliferation. Taken together, these results suggested that miR-148b might function as a tumor-suppressor in NSCLC by regulating cell proliferation and apoptosis.

As we all know that the miRNAs regulated multiple malignant incidences via regulation of specific pathways [38–40]. To further understand the target network involved in the function of miR-148b, we studied the MAPK/JNK signaling pathway. JNK belongs to the MAPK family, and regulates several important cellular functions including cell proliferation, differentiation, survival and apoptosis [41–43]. JNK can be activated by dual phosphorylation of MKK4 and MKK7 [44]. Therefore, we investigated whether miR-148b regulates the tumor growth of non-small cell lung cancer through targeting MAPK/JNK pathway. The results demonstrated that over-expression of miR-148b significantly decreased the protein expression of phosphorylated (p) JNK in NSCLC cells, while the protein expression of MKK4, MKK7 and unphosphorylated (t) JNK was not affected. Our results confirm that miR-148b could inhibit the MAPK/JNK signaling pathway by decreasing the expression of phosphorylated (p) JNK in NSCLC cells. Knocking down miR-148b expression could enhance the phosphorylation of JNK.These results support our previous hypothesis that miR-148b exert anti-cancer activity in NSCLC by targeting MAPK/JNK pathway. In accordance with our study, Fang et al. found that decreased miR-148b could enhance tumor cell proliferation and inhibit apoptosis in human renal cancer cells by targeting MAP3K9, which is a upstream activator of MAPK/JNK pathway [45]. At the same time, several studies have confirmed that miR148b works through the regulation of MAPK/JNK [46, 47].

## Conclusions

In summary, our study demonstrated that miR-148b could significantly inhibit tumor growth. MiR-148b inhibits NSCLC cell proliferation, colony formation, and induces apoptosis through targeting the MAKP/JNK pathway. All of these results suggest that miR-148b could be viewed as a tumor suppressor and might serve as a potential therapeutic candidate in the developing novel strategy for NSCLC patients.

## Abbreviations

GAPDH: Glyceraldehyde 3-phosphate dehydrogenase
miRNAs: MicroRNAs
MKK4: Mitogen activated protein kinase kinase 4
MKK7: Mitogen activated protein kinase kinase 7
MOI: Multiplicity of Infection
NC: Negative control
NSCLC: Non-small cell lung cancer
qRT-PCR: Real time quantitative polymerase chain reaction
SCLC: Small-cell lung cancer
